# Residue Analysis of Insecticides in Potatoes by QuEChERS-dSPE/UHPLC-PDA

**DOI:** 10.3390/foods9081000

**Published:** 2020-07-26

**Authors:** Débora Reis, Pedro Silva, Rosa Perestrelo, José S. Câmara

**Affiliations:** 1 CQM—Centro de Química da Madeira, Campus da Penteada, Universidade da Madeira, 9020-105 Funchal, Portugal; debora_reis26@hotmail.com (D.R.); pedro_dasilva@hotmail.com (P.S.); jsc@staff.uma.pt (J.S.C.); 2 Departamento de Química, Faculdade de Ciências e Engenharia, Campus da Penteada, Universidade da Madeira, 9020-105 Funchal, Portugal

**Keywords:** insecticides, potatoes, full factorial design, QuEChERS-dSPE, UHPLC-PDA

## Abstract

Insecticides are broadly applied in agriculture to defend crops from illnesses and pest attacks, consequently guaranteeing high production. However, their residual deposits in food products are becoming a main concern with regard to human consumption. As such, sensitive analytical methods should be developed to assess, prevent and control insecticide residues. In this research, an accurate, fast and reliable residual analytical method, that is quick, easy, cheap, effective, rugged and safe, combined with dispersive solid phase extraction (QuEChERS-dSPE), was developed for the determination of the most common insecticides used in potatoes cultivation (chlorpyrifos, λ-cyhalothrin, deltamethrin and acrinathrin), using an ultra-high performance chromatography photodiode array detector (UHPLC-PDA). The most influential extraction and instrumentation parameters that affect the method’s performance, such as extraction solvent, ratio salts, sorbents, stationary phases, gradient conditions and eluents, were assessed. Under the ideal conditions, good linearity (0.992–0.998), limits of detection (0.02–0.47 µg/kg) and quantification (0.06–1.58 µg/kg), recovery (94.1 to 112%) and precision (relative standard deviation <18%) were achieved for spiked levels between 2.5 and 50 µg/kg. The obtained results revealed that the potatoes analyzed do not represent any concern for human healthy, as the insecticide residues detected were lower than the maximum residue limits set by the European Union, Codex Alimentarius, and other organizations.

## 1. Introduction

Potatoes (*Solanum tuberosum* L.; *Solanaceae* family) are one of the most representative and widely grown food crops in the world, after rice and wheat, related to human intake. They can grow in any climatological condition, taking less time to grow than other vegetables (e.g., carrots, beets, etc.). Aside from being high in water when fresh, potatoes are primarily composed of carbohydrates and contain moderate amounts of protein and fibers (pectin, cellulose and hemicellulose). Moreover, they are rich in bioactive compounds, namely polyphenols, of which chlorogenic acid, catechin and lutein are the most predominant. In addition, potatoes constitute a good source of several vitamins, ascorbic acid and vitamin B6 being the main vitamins found in potatoes, as well as minerals, particularly potassium. The biggest producer of potatoes worldwide is China, with about one third of the world’s potatoes produced in China and India. In 2018, over 368 million metric tons of potatoes were produced worldwide, according to Food and Agriculture Organization (FAO) estimates. Nevertheless, its production is influenced by numerous pests, causing severe harms to plants and therefore significant yield reductions. In order to control a diversity of pests and defend crops and agricultural commodities, farmers resort to the use of pesticides [1]. There is a wide variety of pesticides, categorized according to their type of use. Herbicides, the main group of pesticides, are used to exterminate weeds and other plants growing in locations where they are unwelcome, whereas insecticides and fungicides are used to exterminate insects and fungi, respectively. Other types include acaricides, molluscicides, nematicides, pheromones, plant growth regulators, repellents and rodenticides. These pesticide classes may be used on the soil to exterminate soil-borne pests, or on the aerial part of the plant [1]. In the cultivation of potatoes, insecticides, namely organophosphorus (e.g., chlorpyrifos) and *pyrethroids* (e.g., λ-cyhalothrin, deltamethrin, acrinathrin), are widely used to protect crops from pest and disease attacks [2,3,4]. In spite of their effectiveness in agricultural application, the imprudent application of insecticides in processing, storage, transport and/or marketing can contribute to their harmfulness, which involves the emission of metabolites and degradation products into the environment (e.g., soil, water, foods) [5,6]. Insecticide presence has been connected to a varied spectrum of human health vulnerabilities, ranging from headaches and nausea, to chronic impacts, endocrine system disruption, reproductive harm, and even cancer. In addition, inappropriate use of insecticides may cause damage to the environment, increase resistance in the target pest organisms, and have deleterious effects on non-target organisms [7]. Therefore, it is crucial to regulate such contaminants in food, and to determine if they pose any danger to public health and to the environment. In this scenario, to guarantee that levels of insecticide residues on vegetables are safe, they must meet current tolerances or maximum residue limits (MRLs, Table 1), normally in µg/kg, which are strictly set by the European Union (EU), Codex Alimentarius (CA) and other organizations [6,8].

The extraction of insecticides from potatoes is a hard task, due to the low concentration that these targets are often present in. Several extraction procedures have been proposed to extract insecticides from foods, such as solid phase extraction (SPE) [9], liquid–liquid extraction (LLE) [10], magnetic solid phase extraction (MSPE) [11], solid phase microextraction (SPME) [12], supercritical fluid extraction (SFE) [3], ultrasonic-assisted dispersive liquid–liquid microextraction (UADLLME) and stir bar sportive extraction (SBSE) [13]. These extraction procedures are time-consuming and labor-intensive [4]. Nowadays, a quick, easy, cheap, effective, rugged and safe method (QuEChERS), followed by clean-up steps involving dispersive solid phase extraction (dSPE), is the most common extraction procedure for extracting insecticide from food samples [14]. QuEChERS-dSPE allows one to attain a final extract with fewer interferences, since dSPE permits the application of diverse amounts and/or combinations of sorbents [4]. Optimization of extraction solvents, and measurements of amounts of salts, buffers, sorbents, and so on, in the dSPE step, should be done to increase the extraction efficiency [15].

The analysis of insecticide residues in foods requires highly selective and sensitive analytical methods. Current analytical methods include mass spectrometry (MS) or tandem mass spectrometry (MS/MS) detection, coupled to gas (GC) or liquid chromatography (LC) [4,8,16]. Nevertheless, ultra-high-performance chromatography (UHPLC) can be a fascinating substitute in the analysis of insecticide residues, due to its great resolution, reduced analysis time, lower solvent volumes and improved selectivity [17].

The aim of the current research was to optimize and validate a QuEChERS-dSPE extraction procedure, combined with UHPLC-PDA, for the simultaneous quantification of the four most-used insecticides on potatoes cultivated on Madeira Island. QuEChERS-dSPE/UHPLC-PDA optimization, including the partition salts ratio, extractant and sorbent selection and chromatographic conditions adjustment, would contribute to developing a simple, reliable and environmental-friendly analytical method for quantifying insecticides. After the optimization of experimental and instrumental parameters, the analytical method was validated in terms of linearity, selectivity, precision (intra- and inter-days), recovery, limits of detection (LOD) and quantification (LOQ), and applied to different potato varieties cultivated on Madeira Island, to monitor the occurrence and determine the levels of insecticides present in different varieties of potatoes. 

## 2. Materials and Methods

### 2.1. Standards and Materials

All chemicals and solvents were of analytical quality grade. HPLC grade acetonitrile (MeCN) and methanol (MeOH) were purchased from LabScan (Dublin, Ireland). Insecticide standards, chlorpyrifos (98.3%), acrinathrin (99.7%), deltamethrin (99.9%) and ʎ-cyhalothrin (≥95.0%) were supplied by Sigma-Aldrich (St. Louis, MO, USA), Table 1. Buffered salts for QuEChERS extraction, including anhydrous magnesium sulphate (MgSO_4_), sodium hydroxide (NaOH), sodium chloride (NaCl), trisodium citrate dihydrate (C_6_H_5_Na_3_O_7_·2H_2_O) and disodium hydrogencitrate sesquihydrate (C_6_H_8_Na_2_O_8_), were also obtained from Sigma-Aldrich. dSPE clean-up tubes with primary secondary amine (PSA) and MgSO_4_ were obtained from Waters (Milford, MA, USA). Phosphoric acid (PhA, ≥85%) was from BDH (Poole, UK), while formic acid (FA, ≥99%) was obtained from Merck (Darmstadt, Germany). Ultrapure water (H_2_O) from a Milli-Q ultrapure water purification system (Millipore, Bedford, USA) was used for preparing the UHPLC mobile phase and other aqueous solutions. Filters of 13 mm with 0.22-µm PTFE membranes were used for filtration of the final extracts before UHPLC-PDA analysis.

### 2.2. Samples

Potatoes, as with most food matrices, require a preliminary complex and time-consuming stage. To ensure the maximum efficiency of the process, it is preferable to use the smallest amount of sample as long as it guarantees statistical representativeness in the final result. In this case, for obtaining a solid homogeneous sample, 1 kg of potatoes was chopped into small pieces, followed by a lyophilization process. This procedure is normally used on foods that have a high water content (potato is about 80% H_2_O). All samples were kept in the freezer at −20 °C, in the dark until further investigation.

### 2.3. Standard Solutions

Individual insecticide standard solutions (1 mg/L) were prepared in MeCN, labelled, and stored at −20 °C in the dark. Intermediate stock solutions in MeCN, containing 10 mg/L of each insecticide, were prepared by mixing adequate amounts of the individual stock solutions. The working standard solutions, used to construct the calibration curve, were created by dilution of the stock solution with matrix in MeCN, to obtain the concentration range reported in Table 2. Each one of these solutions was analyzed in triplicate, using the QuEChERS-dSPE/UHPLC-PDA method.

### 2.4. Optimization of the Modified QuEChERS-dSPE Extraction Procedure by Experimental Design

The multivariate optimization process of the QuEChERS-dSPE method is a crucial step, due to the low concentration of analytes in the sample, the different chemical properties, and the complexity of matrices. The main purpose of the multivariate optimization process is to increase the extraction efficiency, to concentrate the analytes and to remove untargeted compounds.

The powdered potatoes (1.05 ± 0.01 g) spiked with 20 μg/kg of insecticides (n = 3) were extracted with different extraction solvent percentages (50%, 75% and 100% of MeCN), acidification (without, 0.1% FA and 0.1% PhA) and ratios of partition salts (1:1:0.5:1; 2:1:0.5:1; 3:1:0.5:1 for MgSO_4_:NaCl:C_6_H_8_Na_2_O_8_:C_6_H_5_Na_3_O_7_·2H_2_O).

For optimization of the dSPE clean-up, after the partitioning step, the supernatants (1 mL) were moved into diverse dSPE sorbent combinations: (i) 150 mg MgSO_4_, (ii) 150 mg MgSO_4_ + 25 mg PSA, (iii) 150 mg MgSO_4_ + 25 mg C_18_ and (iv) 150 mg MgSO_4_ + 25 mg PSA + 25 mg C_18_. Moreover, the ultrasound extraction time (0, 15 and 30 min), as well as the final extract pre-concentration, were also evaluated.

The optimal QuEChERS-dSPE extraction efficiency was chosen through a full factorial design process, which involves combining all the possible variables and studying the effects of each factor on the response variable, as well as the interaction effects between the factors. All the experiments were done in triplicate, and the ideal extraction conditions were selected according to total peak area and reproducibility, expressed as relative standard deviation (% RSD).

#### Optimized QuEChERS-dSPE Procedure

**Figure 1:**
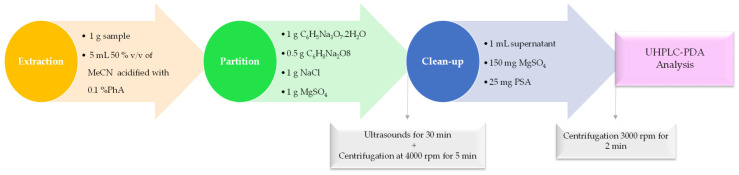
Schematic representation of the quick, easy, cheap, effective, rugged and safe followed by dispersive solid phase extraction (QuEChERS-dSPE) procedure.

For the QuEChERS procedure (Figure 1), 1 g of potatoes was weighed to the accuracy of 0.01 g, then was put into a 10-mL polytetrafluoroethylene (PTFE) centrifuge tube, mixed and left to stand for 15 min at room temperature before the extraction. Then, 5 mL of 50% (*v*/*v*) MeCN acidified with 0.1% PhA was added to the tube, and the mixture was shaken energetically for 1 min with a vortex guaranteeing that the solvent interrelated well with the entire sample. After that 1 g of C_6_H_5_Na_3_O_7_·2H_2_O, 0.5 g of C_6_H_8_Na_2_O_8_, 1 g of NaCl and 1 g of MgSO_4_ were added to the homogenized mixture, and mixed in a vortex for 2 min and submitted to ultrasound for 30 min, followed by a centrifugation at 4000 rpm for 5 min at 25 ± 1 °C, completing the partition step and the consequent separation of phases (H_2_O and MeCN phase). Next, to promote the clean-up, 1 mL of the supernatant was transferred to a 2-mL PTFE dSPE clean-up tube containing 150 mg MgSO_4_ and 25 mg of PSA. The mixture was centrifuged (4000 rpm, 5 min, 25 °C) and 400 μL of upper phase was filtered through a 0.22-µm PTFE filter membrane, and transferred into a vial prior to the UHPLC-PDA analysis.

### 2.5. Optimization of UHPLC-PDA Conditions

The optimal separation conditions for UHPLC analysis were also chosen through a full factorial design. Different factors were combined and studied, namely different eluents (H_2_O, H_2_O acidified with 0.1% of FA or 0.1% of PhA), column flows (150, 250 and 350 µL/min) and column temperature (30, 40 and 50 °C). All the conditions were tested the same way, in triplicate in the four available analytical columns, namely CORTECS (90 Å, 2.1 mm × 100 mm, 1.6 μm), BEH (130 Å, 2.1 mm × 100 mm, 1.7 μm), HSS (100 Å, 2.1 mm × 100 mm, 1.8 μm) and CSH (130 Å, 2.1 mm × 50 mm, 1.7 μm).

#### Optimized UHPLC-PDA Conditions

The separation, identification and quantification of insecticide residues was performed on a Waters Ultra Pressure Liquid Chromatographic Acquity system (UPLC, Acquity H-Class) (Milford, MA, USA) combined with a Waters Acquity quaternary solvent manager (QSM), an Acquity sample manager (SM), a column heater, a 2996 PDA detector and a degassing system. The whole configuration was driven by Empower software v2.0 from Waters Corporation.

The separation was performed in a CORTECS column (2.1 mm × 100 mm, 1.8 μm) using H_2_O acidified with 0.1% PhA (A) and MeCN (B) as mobile phase, with a flow rate of 150 µL/min. The following gradient program was used: an initial composition of 95% phase A for 2 min; an increase in phase B to 75% from 2.0 to 29.0 min; a decrease of phase B to 5% from 29 to 30 min, and further column re-equilibration from 30 to 33 min, given a total run time of 30 min. The injection volume and the column temperature were 10 µL and 30 °C, respectively. After each injection the needle was washed firstly with 400 µL of H_2_O:MeCN (90:10, *v*/*v*) and after with 200 µL of H_2_O:MeCN (10:90, *v*/*v*) solution. The samples were preserved at 15 °C during the analysis.

For quantification purposes, the PDA detection was performed by applying different channels that were set to the extreme absorbance wavelengths of each insecticide residue (190–310 nm). The target was identified by relating the retention time (RT) and spectral features of their peaks with those of the standards. The quantification was carried out by means of the standards of each insecticide, in triplicate. The results were presented as mean ± standard deviation.

### 2.6. Method Validation

The method validation was performed to certify that the method was accurate, precise, reproducible and robust for the indicated range in which an analyte will be examined. For this purpose, the validated method should include assays related selectivity, linearity, accuracy, precision, matrix effect, limit of detection (LOD) and limit of quantification (LOQ), according to European Union SANCO/12495/2011 guidelines. The selectivity was evaluated by the lack of interfering peaks at the analyte RT. This parameter refers to the level to which a method can quantify a specific analyte in a complex mixture without interference from other compounds.

Linearity is the ability of the method to verify if the samples solutions are within a concentration range where the analyte response is directly proportional to the concentration. So, the method’s linearity was evaluated in the concentration range reported in Table 2, based on the average peak areas vs. concentrations, % RSD, coefficient correlation (R^2^) and linear ranges established for each analyzed insecticide.

The sensitivity of the method was assessed through LOD and LOQ. The LOD is defined as the lowest concentration of analyte that can be detected, but not necessarily quantitated. On the other hand, LOQ is frequently defined as the lowest amount of analyte that can be determined quantitatively with acceptable precision and accuracy, under the stated operational conditions of the method. The LOD and LOQ were calculated by the multiplications by 3 and 10 of the ratio of standard deviation(s) of calibration curve interception, and the slope of a regression curve.

The precision of an analytical method can be defined as the closeness of agreement between the values attained under specified conditions. Precision was determined at two different levels: repeatability (intra-run precision) and intermediate precision (inter-run precision). Repeatability is achieved with the same method under equal conditions, in the same laboratory by the same analysts using the same equipment within a short period of time. The assessment of intermediate precision is attained with the same method, under similar conditions in the same laboratory, but by diverse operators, using different equipment over an extended interval of time. The precision is expressed as % RSD.

Accuracy is the nearness of agreement among the experimental values attained by the analytical method and the true value. The accuracy was evaluated through percentage of recovery. The recovery was attained by relating the known theoretical concentration added to the sample (C_theoretical_) to the experimental concentration (C_experimental_) of each insecticide in the sample, spiked at low (LL, 2.50 µg/kg), medium (ML, 25.0 µg/kg) and high (HL, 50.0 µg/kg) concentration levels. The C_experimental_ was determined via the variance between the peak areas of the analytes in spiked and non-spiked samples.

The matrix effect is most pronounced in complex sample analyses. The matrix effect was calculated using the method of “standard additions” to a sample under study, which was assessed via the association of the slopes attained from the calibration curves of the insecticides in a sample and the solvent-based matrix.

## 3. Results and Discussion

The improvement of a reliable analytical method, linked to simplicity and speed, is still a difficult task. Thus, numerous parameters should be optimized, and problems can mainly be found particularly in the sample preparation step. All optimized parameters were carried out in triplicate, using potatoes spiked with a mixture of insecticides at the concentration of 20 μg/kg as the matrix.

### 3.1. Optimization of QuEChERS-dSPE Procedure

The difficulty of sample treatment is connected to possible matrix interferences, as well as the physicochemical properties of the analyte. The selection of the extraction solvent is one of the fundamental points in the development of an insecticide extraction procedure. Many aspects should be considered, among them: the capacity to extract a widespread spectrum of pesticides with dissimilar polarities; selectivity for compounds of interest during extraction, partition and clean-up; compatibility with diverse chromatographic techniques; and economical, safety and environmental sustainability.

**Figure 2:**
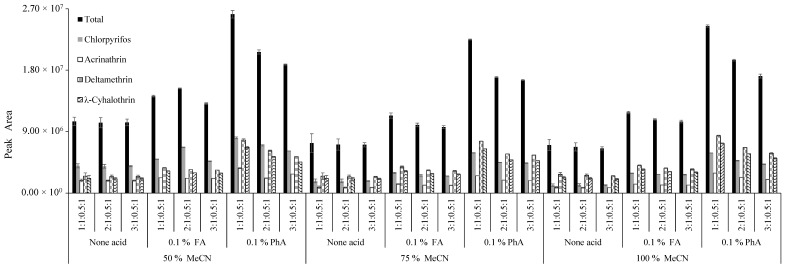
Influence of acetonitrile (MeCN), acidification (none, 0.1% FA and 0.1% PhA) and partition salts ratio in the extraction step for each studied insecticide. Abbreviations—FA: formic acid; PhA: phosphoric acid.

Ethyl acetate, acetone and MeCN are the most common solvents used to extract pesticides via multiresidue methods [14]. Ethyl acetate has been shown to be a solvent with widespread features, since it has the capacity to extract pesticides of different classes in several types of samples. However, the recovery rates of some pesticides are small, due to degradation problems. In those cases, the addition of NaOH is necessary for an increase of recovery percentages [14]. Acetone and MeCN are miscible with water, and promote extraction in a single phase when in interaction with the matrix. When an extraction is carried out with acetone, there is a need to add non-solvents so that the parting between the organic and aqueous phases occurs. However, this is not required when MeCN is used, since the addition of salts to the extract causes such separation [18]. Meanwhile, the use of MeCN permits the extraction of a small number of lipophilic co-extractives from the sample (e.g., waxes, fats, pigments), and facilitates the extraction of a wide range of pesticides with dissimilar polarities and with higher capacity and selectivity [18]. Another great advantage is that MeCN is more suitable for liquid chromatography (LC) than acetone and/or ethyl acetate. Thus, MeCN was the solvent selected to extract the target insecticides. In addition, to guarantee the best extraction efficiency and to reduce hazardous organic solvent consumption as much as possible, different percentages of MeCN in aqueous solution (50, 75 and 100%) were evaluated. Its acidification was also tested to facilitate the extraction of a wide range of pesticides with different polarities (FA and PhA), and lastly, the addition of partition salts ratio in the partition step (1, 2 or 3 mg MgSO_4_), once the hydration of MgSO_4_ has been reported, results in a high exothermic process, causing the sample extract to get hot during the extraction/partition step, reaching temperatures as high as 40–45 °C. The heat is beneficial for extraction, especially in the case of less polar pesticides [19]. The results are exhibited in Figure 2. Overall, better extractions were achieved with the lowest percentage of MeCN (50%), which provided higher total peak areas for most studied insecticides. The total peak areas of insecticides declined in the subsequent order of MeCN percentages in the extraction solution: 50 > 75 > 100%. This is in agreement with Correia-Sá et al. [20], who observed an increase in recovery percentage for all analyzed pesticides (recoveries ranged from 77% to 130% with the addition of H_2_O, and from 20% to 46% without H_2_O addition). Years later, a study directed by Frenich et al. [21] utilized an extraction mixture consisting of MeCN:H_2_O (80:20, *v*/*v*) and sonication. The method was able to extract most analytes successfully, with good recoveries. Additionally, according to the obtained results, as expected from the literature, the extraction of pesticides with 100% MeCN was not efficient enough to reduce the lowest values of the peak area [20].

As can be observed in Figure 2, the acidification with 0.1% FA or PhA increased the extraction efficiency of insecticides in all different conditions. This is in agreement with the literature, since acidified MeCN promoted the adequate recovery of pesticides, which generally present stability problems and also minimize degradation [22]. The acid addition also showed a great influence not only on the peak areas, but also on their shapes. The enhanced total peak areas were achieved when using the inorganic acid, due to its higher capacity to protonate analytes and reduce hydrophilicity [22]. Therefore, MeCN with 0.1% of PhA was chosen as the optimal extraction solvent.

Concerning the use of salts to promote phase separation, the 1:1:0.5:1 ratio of MgSO_4_:NaCl:C_6_H_8_Na_2_O_8_:C_6_H_5_Na_3_O_7_·2H_2_O was shown to be the most effective in terms of selectivity and ability to separate aqueous and organic phases, preserving high peak area values and low co-extractions of interferences in all assays. Other ratios did not prove to be so effective, maybe because of the effect of temperature on the extraction of thermally-labile analytes. Some pesticides can be degraded with the addition of MgSO_4_ anhydrous, due to its exothermic hydration reactivity [23]. The combination of other salts, such as NaCl, also helping to promote the salting-out effect, consequently improved the extraction efficiency of pesticides that frequently induce stability harms [21,22]. For these reasons, 50% of MeCN, acidified with 0.1% PhA and a 1:1:0.5:1 ratio of MgSO_4_:NaCl:C_6_H_8_Na_2_O_8_:C_6_H_5_Na_3_O_7_·2H_2_O, was selected for the further assays.

**Figure 3:**
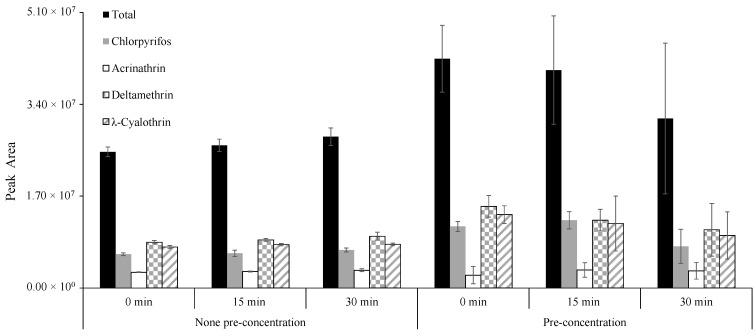
Influence of ultrasound time and pre-concentration until dryness on insecticides extraction.

Ultrasound-assisted extraction (UAE) is considered a suitable substitute for pesticide extraction from several diverse matrices, including vegetables [24]. Therefore, this extra-step was added to the traditional QuEChERS procedure in order to attain higher extraction efficiency. For this purpose, diverse ultrasound extraction times were tested (0, 15 and 30 min). Moreover, the preconcentration step, involving evaporating the extract till dryness under a stream of N_2_, was also considered, in order to enrich the analyte before UHPLC-PDA analysis. The obtained results are represented in Figure 3.

According to the obtained results, it was possible to observe that the preconcentration slightly increased the extraction efficiency, but this additional step did not show coherent and satisfactory results once high RSD values (>35%) were obtained, compared to no preconcentration. This result may be explained by the precipitation of matrix co-extractives occurring during the step of evaporation till dryness. In the analysis of the no-preconcentration-step method, the UAE led to very pleasing results by increasing the extraction efficiency. It was possible to observe a relation between different ultrasound times and extraction efficiencies, the better extraction efficiency being obtained with 30 min of UAE extraction time. In fact, it was already expected that the increase of both pressure and temperature improves the penetration, transport, solubility and diffusivity of the extraction solvent, resulting in a better extraction of pesticides [25]. It decreases the co-extracted compounds, with consequent reduction or elimination of the required time-consuming clean-up steps [26]. Thence, according with the obtained results, 30 min of UAE without preconcentration was selected.

**Figure 4:**
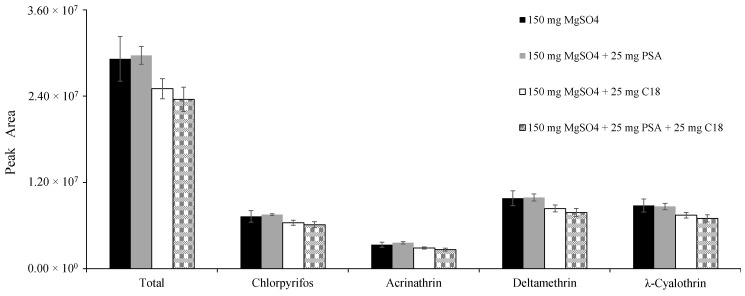
Influence of commonly used clean-up salts, evaluated for their effectiveness on insecticides analysis.

After extraction solvent, salts ratio, UAE extraction and preconcentration were optimized, as above, several sorbent combinations were evaluated for optimum clean-up. The clean-up step is crucial to ensure the robustness and reliability of the results attained by the chromatographic system [27]. Due to the presence of co-extractives in the extracts, it became necessary to evaluate the effectiveness of the most commonly used chemicals for this step. For this reason, the extraction efficiencies of MgSO_4_, MgSO_4_ + PSA, MgSO_4_ + C_18_ and MgSO_4_ + PSA + C_18_ were evaluated. MgSO_4_ is used to isolate water from the organic solvent; on the other hand, PSA removes many organic acids, polar pigments, some sugars and fatty acids. Finally, C_18_ eliminates non-polar-interfering substances [28]. The results are presented in Figure 4.

The cleaner chromatogram and enhanced total areas were obtained using MgSO_4_ + PSA. The elimination of residual water and clean-up were carried out simultaneously using dSPE, in which 150 mg MgSO_4_ and 25 mg PSA sorbent are mixed with the MeCN extract. Together, they effectively remove many polar-interfering components from the potato extract [27,29]. Combining C_18_ with the clean-up procedure did not positively influence the cleaning of the extracts, because it is normally used for fatty matrices (e.g., milk, eggs, olive oil, avocado), efficiently removing interfering substances in those cases. However, it has been reported that it also removes definite pesticides, like thiabendazole and other planar-ring pesticides [28]. Thus, 150 mg MgSO_4_ + 25 mg PSA was selected for the clean-up step.

### 3.2. Optimization of UHPLC-PDA Conditions

**Figure 5:**
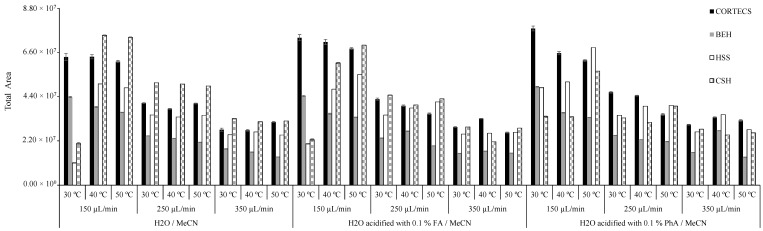
Optimization of chromatographic conditions in different analytical columns.

Chromatographic conditions were assessed to achieve a suitable compromise between separation, resolution and analysis time. A full factorial design was applied to evaluate the following parameters: elution solvent (H_2_O acidified with 0.1% of FA or 0.1% of PhA), column flow (150, 250 and 350 µL/min) and column temperature (30, 40 and 50 °C) for each column under study, namely the CORTECS, BEH, HSS, CSH and HILIC. The results are presented in Figure 5.

Chromatographic efficiency varied significantly depending on column type. The insecticides total peak areas decreased in the following order: CORTECS > CSH > HSS > BEH > HILIC. The high efficiency of CORTECS is elucidated by the small particle core-shell diameter (1.6 µm), which is smaller than that of BEH, HSS and CSH (from 1.7 up to 1.8 µm), which increases the surface absorption and the retention of analytes. These separations were based on the reverse-phase through the C_18_ surface. Moreover, HILIC values are not presented due to their poor results. These can be explained by the fact that a silica phase column was applied in the hydrophilic interaction to facilitate the retention of polar compounds. The two most used kinds of eluents are the organic (e.g., MeOH, MeCN) and aqueous phase, with the addition of some additives [30]. The tested eluents interfered mostly with the peak resolution. 

The addition of same acids during the extraction, specifically FA and PhA, improved peak resolutions for most insecticides. 

From the results, it is possible to confirm that greater total peak areas were a result of column flow rate. Improved results were attained with the lowest flow rate, namely 150 µL/min, whereas the flow rates of 250 and 350 µL/min directly influenced the reduction of resolution and total peak areas in all the studied columns. Furthermore, it was possible to observe that different tested temperatures were not determinant of enhanced results. Small differences were observed between the tested temperatures (30, 40, 50 °C), 30 °C offering slightly better results. Accordingly, the optimum instrumental parameters for improved results were obtained with the CORTECS column at 30 °C, using MeCN and H_2_O acidified with 0.1% PhA as eluents, and a flow rate of 150 µL/min.

### 3.3. Method Validation

The performance of the QuEChERS-dSPE/UHPLC-PDA analytical method was assessed for selectivity, linearity, precision (intra- and inter-day), accuracy (% recovery) and sensitivity (LOD and LOQ), as designated in Section 2.6. The results obtained are presented in Table 2 and Table 3.

**Figure 6:**
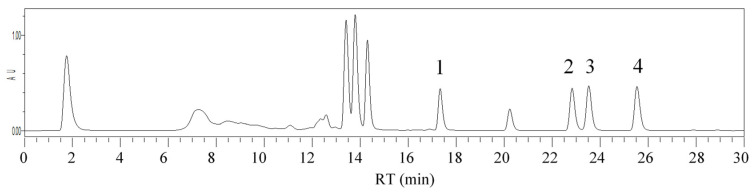
QuEChERS-dSPE/UHPLC-PDA chromatogram at 224 nm for a potato matrix spiked with insecticide residues at 20 µg/kg. Peak identification: 1. Chlorpyrifos; 2. λ-Cyhalothrin; 3. Deltamethrin; 4. Acrinathrin.

The selectivity was evaluated via the nonappearance of interfering peaks at the RT and extreme wavelengths (λ_max_) of the studied insecticides. The evaluation was carried out by checking the peaks of potatoes spiked with target insecticides at quantities of 20 μg/kg, using the optimized QuEChERS-dSPE/UHPLC-PDA procedure. As can be observed in Figure 6, no significant interference was found in the RT or λ_max_ of each insecticide under study in the potato matrix, thus validating the selectivity of the method.

The linearity of the method was assessed using calibration curves that were fit using least square linear regression analysis. The obtained correlation coefficient (R^2^) was higher than 0.992, with residuals not exceeding ±15%, which indicates the method’s linearity over the whole range of concentration investigated, as shown in Table 2. In addition, the developed method shows a very pleasing capability to detect and quantify the insecticides under study. The LOD ranged between 0.02 (deltamethrin) and 0.47 (chlorpyrifos) µg/kg, and the LOQ ranged between 0.06 (deltamethrin) and 1.58 (chlorpyrifos) µg/kg. It is notable that most of the analyzed insecticides can be calculated, with great reliability, at trace-level concentrations (1.58 µg/kg), which is noticeably below their MRLs (Table 1).

The precision and recovery were evaluated by spiking the insecticides in potatoes at different levels of concentration (low, medium and high) (Table 3), in the linear interval of the analytical curve. The intra-day precision varied from 0.90% to 15.7%, while the inter-day precision from 1.02% to 17.1%. The recovery of insecticides ranged from 94.1% to 112%. According to the literature, a quantitative method should be validated as being able to present mean recoveries from 70% to 120%, and its precision should show % RSD values lower than 20%.

Moreover, the analytical method developed was linked with other gas (GC) and liquid chromatography (LC) methods reported in the literature for pesticide quantification in vegetables, Table 4 [1,3,5,8,12,16,31,32,33,34,35]. The sample amount (g), LOD, LOQ and recovery (analytical performance) were considered to demonstrate the advantages of the QuEChERS-dSPE/UHPLC-PDA method here validated. Overall, the current analytical methods proposed here used the lower sample amount (1 g), with the exception of the UADLLME/HPLC-UV method [31]. Nevertheless, the UADLLME/HPLC-UV method requires large volumes of solvents. QuEChERS-dSPE/UHPLC-PDA showed the same, or an enhanced, analytical performance, compared to most of the reference methods. Furthermore, QuEChERS-dSPE/UHPLC-PDA is more economical and environmentally friendly, compared to QuEChERS-dSPE/LC-MS/MS.

### 3.4. Quantification of Insecticides in Potatoes

In order to evaluate the feasibility of the validated method, 10 samples of potatoes were analyzed. Residues of insecticides in the samples analyzed using the QuEChERS-dSPE/UHPLC-PDA method were not detected, or were below the MRLs. Among the 10 samples, chlorpyrifos was found in 3 samples at a mean concentration ranging from 4.76 ± 0.10 to 9.85 ± 0.87 µg/kg, whereas λ-cyhalothrin was found in one sample at a concentration of 6.41 ± 0.02 µg/kg. It is crucial to monitor the insecticide residues in potatoes to guarantee food safety, and consequently, population health.

## 4. Conclusions

The developed and validated QuEChERS-dSPE/UHPLC-PDA method demonstrated that it is a simple, celeritous and appropriate strategy for the identification and quantification of insecticide residues in potatoes.

After optimizing several variables that affect QuEChERS-dSPE performance, the optimal extraction efficiencies were obtained with 50% of MeCN acidified with 0.1% PhA, 1:1:0.5:1 ratio of MgSO_4_:NaCl:C_6_H_8_Na_2_O_8_:C_6_H_5_Na_3_O_7_·2H_2_O, 30 min of ultrasound time and 150 mg MgSO_4_ + 25 mg PSA.

After careful selection of the chromatographic conditions, it was verified that the separation of insecticides with different properties could be achieved with good chromatographic resolution. In addition, PDA has proven to be an economical alternative when compared to more sophisticated and expensive equipment, such as the MS detector, making this method more accessible to other laboratories. The optimal chromatographic conditions were observed using CORTECS with a gradient program combining H_2_O acidified with 0.1% PhA and MeCN, and a flow rate of 150 µL/min at 30 °C column temperature.

Under the optimal conditions, the validated method showed reliable results in terms of selectivity for the studied insecticides, and in terms of linearity, by presenting correlation coefficients (R^2^) higher than 0.992. The LOD ranged from 0.02 (deltametrin) to 0.47 (chlorpyrifos) µg/kg, whereas the LOQ ranged from 0.06 (deltametrin) to 1.58 (chlorpyrifos) µg/kg. Moreover, the obtained results for accuracy ranged between 94.1% and 112%, while in precision, the RSD values were lower than 18%.

The QuEChERS-dSPE/UHPLC-PDA revealed a suitable routine practice since it is simple, economical, precise, accurate and environmentally friendly. In addition, the concentration of insecticides in potatoes was lower than the maximum residue limits (MRLs) set by the European Union (EU), Codex Alimentarius (CA) and other organizations. So, the potatoes analyzed no represent any risk of human exposure via consumption.

## Figures and Tables

**Table 1:** Physicochemical parameters of the investigated insecticides.

		Structure	Chemical Family	MW (g/mol)	Water Solubility 25 °C (mg/L)	LD_50_ (mg/kg mouse)	ADI (µg/kg) bw/day	MRL (µg/kg)	Regulation (EC) Nº
Insecticides	Chlorpyrifos C_9_H_11_Cl_3_NO_3_PS	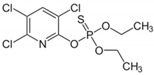	Organophosphorus	350.57	2	32–1000	1	50	839/2008
	Acrinathrin C_26_H_21_F_6_NO_5_	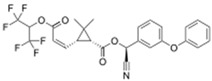	Pyrethroid	541.45	0.02	5000	10	50	839/2008
	Deltamethrin C_22_H_19_Br_2_NO_3_	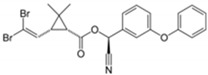		505.21	<0.002	5000	10	300	2016/1822
	λ-Cyhalothrin C_23_H_19_ClF_3_NO_3_	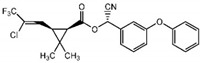		449.85	0.005	56	2.5	20	834/2013

MW—molecular weights; LD_50_—median lethal doses; ADI—acceptable dose intake; MRL—maximum residue level; EC—European Council Directive.

**Table 2:** Parameters of calibration, limit of detection (LOD), limit of quantification (LOQ) and matrix effect (%) for insecticides using QuEChERS-dSPE/UHPLC-PDA method.

RT (min)	Insecticides	λ_max_ (nm)	Concentration Range (µg/kg)	Regression Equation	R^2^	LOD (µg/kg)	LOQ (µg/kg)	ME (%)
17.55	Chlorpyrifos	197	2.5–50	y = 980.04x − 92.661	0.994	0.47	1.58	93
23.25	λ-Cyhalothrin	190	2.5–50	y = 17,590x − 24,725	0.998	0.17	0.57	110
23.99	Deltamethrin	191.4	2.5–50	y = 176,281x − 779,412	0.992	0.02	0.06	100
26.28	Acrinathrin	192	2.5–50	y = 1159x + 1021.3	0.998	0.13	0.44	86

RT: retention time; R^2^: regression coefficients; LOD: limit of detection: LOQ: limit of quantification; ME; Matrix effect.

**Table 3:** Recovery and precision of insecticides in potatoes at three spiked levels.

Insecticides	Concentration Range (µg/kg)		Precision (% RSD)		Accuracy
	Theoretical	Experimental	Intraday	Interday	Rec (%) ± SD
Chlorpyrifos	2.50	2.7	15.0	14.6	106 ± 3.46
	25.0	23.5	7.65	2.80	94.1 ± 3.82
	50.0	52.1	7.62	2.10	104 ± 4.19
λ-Cyhalothrin	2.50	2.6	12.7	7.89	105 ± 3.14
	25.0	25.7	2.87	5.99	103 ± 4.08
	50.0	49.7	0.92	1.49	99.4 ± 4.56
Deltamethrin	2.50	2.80	10.8	13.6	112 ± 2.43
	25.0	26.7	6.72	3.38	107 ± 3.84
	50.0	49.7	0.90	1.02	99.4 ±3.37
Acrinathrin	2.50	2.20	10.6	9.47	87.2 ± 4.26
	25.0	23.6	15.7	17.1	94.4 ± 1.81
	50.0	51.5	5.10	3.20	103 ± 0.97

RT—Retention time; REC (%)—Recovery percentage; SD—Standard deviation.

**Table 4:** Analytical parameter comparison of several studies carried out for pesticides quantification in vegetables.

Sample (Amount, g)	Extraction Procedure	Analytical Method	LOD (µg/kg)	LOQ (µg/kg)	Rec (%)	Ref
Vegetables (2)	QuEChERS	GC-MS	-	2–49.6	70–114	[32]
Fruits/Vegetables (10)	QuEChERS-DLLME	GC-MS	1.0–10	5.0–34	87–106	[5]
Vegetables (5)	QuEChERS-dSPE	GC-MS/MS	-	<10	70–120	[8]
Fruits/Vegetables (5)	HS-SPME	GC-ECD	0.11–0.23	0.37–0.77	81–106	[12]
Fruits/Vegetables (10)	QuEChERS-dSPE	LC-MS/MS	0.1–1	0.5–5	77–110	[16]
Fruits/Vegetables (10)	QuEChERS-dSPE	UHLC-MS/MS	0.003–2.0	0.01–6.67	73–134	[33]
Potatoes (10)	QuEChERS-dSPE	UHLC-MS/MS	0.4–1.0	2.0–5.0	81–113	[1]
Fruits (0.5)	UADLLME	HPLC-UV	1.15–2.46	4.38–6.16 *	83–91	[31]
Fruits/Vegetables (1)	SFE-MSPE	HPLC-UV	-	100	91–99	[3]
Vegetables (2)	MSPD	HPLC-UV	20	70	87–105	[34]
Potatoes (10)	QuEChERS-dSPE	HPLC-DAD	0.9	2.7	86–90	[35]
Potatoes (1)	QuEChERS-dSPE	UHPLC-PDA	0.02–0.47	0.44–1.58	94–112	This work

Abbreviations—GC-ECD: gas chromatography equipped with an electron capture detector; GC-MS/MS: gas chromatography tandem mass spectrometry; GC-MS: gas chromatography coupled with mass spectrometry; HPLC-DAD: high-performance liquid chromatography with a diode-array detector; HPLC-UV: high-performance liquid chromatography with ultraviolet detector; HS-SPME: headspace solid phase microextraction; LOD: limit of detection; LOQ: limit of quantification; MSPD: matrix solid-phase dispersion; QuEChERS: quick, easy, cheap, effective, robust and safe; QuEChERS-DLLME: quick, easy, cheap, effective, robust and safe followed by dispersive liquid–liquid microextraction; QuEChERS-dSPE: quick, easy, cheap, effective, rugged and safe technique coupled with dispersive solid-phase extraction; Rec (%): recovery; SFE-MSPE: Supercritical fluid extraction followed by magnetic solid phase extraction; UADLLME: ultrasonic-assisted dispersive liquid–liquid microextraction; UHLC-MS/MS: ultra-high performance liquid chromatography coupled with tandem mass spectra; UHPLC-PDA: ultra-high performance liquid chromatography coupled with photodiode array detector. * Expressed as µg/L.
